# The Role of 39 Psoriasis Risk Variants on Age of Psoriasis Onset

**DOI:** 10.1155/2013/203941

**Published:** 2013-09-23

**Authors:** Yingchang Lu, Sinae Kane, Haoyan Chen, Argentina Leon, Ethan Levin, Tien Nguyen, Maya Debbaneh, Jillian W. Millsop, Rishu Gupta, Monica Huynh, Daniel Butler, Kelly Cordoro, Wilson Liao

**Affiliations:** ^1^Department of Dermatology, University of California San Francisco, 2340 Sutter Street, P.O. Box 0808, San Francisco, CA 94143-0808, USA; ^2^Department of Gastroenterology, Ren Ji Hospital, Shanghai Jiao Tong University School of Medicine, Shanghai Institute of Digestive Diseases, Shanghai 200001, China; ^3^University of California Irvine School of Medicine, Irvine, CA 92697, USA; ^4^University of Utah School of Medicine, Salt Lake City, UT 84132, USA; ^5^Keck School of Medicine, University of Southern California, Los Angeles, CA 90089, USA; ^6^Chicago College of Osteopathic Medicine, Downers Grove, IL 60515, USA

## Abstract

Recent genome-wide association studies (GWAS) have identified multiple genetic risk factors for psoriasis, but data on their association with age of onset have been marginally explored. The goal of this study was to evaluate known risk alleles of psoriasis for association with age of psoriasis onset in three well-defined case-only cohorts totaling 1,498 psoriasis patients. We selected 39 genetic variants from psoriasis GWAS and tested these variants for association with age of psoriasis onset in a meta-analysis. We found that rs10484554 and rs12191877 near *HLA-C* and rs17716942 near *IFIH1* were associated with age of psoriasis onset with false discovery rate < 0.05. The association between rs17716942 and age of onset was not replicated in a fourth independent cohort of 489 patients (*P* = 0.94). The imputed *HLA-C∗06:02* allele demonstrated a much stronger association with age of psoriasis onset than rs10484554 and rs12191877. We conclude that despite the discovery of numerous psoriasis risk alleles, *HLA-C∗06:02* still plays the most important role in determining the age of onset of psoriasis. Larger studies are needed to evaluate the contribution of other risk alleles, including *IFIH1*, to age of psoriasis onset.

## 1. Introduction

Psoriasis is an inflammatory, immune-mediated disorder of the skin, joints, and nails with an estimated prevalence of 2-3% of the population. Henseler and Christophers divided psoriasis into two subtypes. Type I psoriasis manifests before age 40 with peak onset at 16–22 years, and Type II psoriasis begins after age 40 with peak onset at 57–60 years [1]. Type I and II psoriasis have been shown to differ clinically in their severity, relapse frequency, and family history [1, 2]. The clinical differences in Type I and Type II psoriasis are paralleled by genetic differences. Type I psoriasis has a stronger genetic basis as a greater proportion of patients had a family history of psoriasis, and has stronger *HLA-C∗06* associations; however, Type II psoriasis is negative in family history, and is not associated with *HLA-C∗06* [1, 3, 4]. Over the past few years, over 36 novel psoriasis loci have been identified through genome-wide association studies (GWAS) [5–13]. However, it is not known to what degree these susceptibility alleles influence the age of onset of psoriasis. Here, we examined 39 of these genetic variants in 3 cohorts of psoriasis cases to ascertain whether any of these loci were preferentially associated with the age of psoriasis onset. 

## 2. Materials and Methods

The SNPs were selected from psoriasis GWAS conducted in Caucasian populations (published before December 2012; see Supplementary Table 1 available online at http://dx.doi.org/10.1155/2013/203941), taking into account the potential molecular mechanisms underlying the pathogenesis of psoriasis [14]. We evaluated 39 SNPs for association with age of psoriasis onset in two psoriasis GWAS case cohorts; 13 of those SNPs were genotyped by Illumina GoldenGate assay in a third cohort of 418 cases at the University of California San Francisco (UCSF) (Table 1). rs17716942 in *IFIH1* was genotyped in a fourth independent cohort of 489 National Psoriasis Foundation (NPF) cases using the Applied Biosystems Taqman genotyping assay. Age of onset of psoriasis was determined by patient self-report and by the treating dermatologist. The two GWAS cohorts consisted of the Genetic Association Information Network (GAIN) cohort including 898 cases [7] and the Washington University/University of California San Francisco cohort (WashU/UCSF) including 182 cases [6]. IMPUTE2 [15] was used to impute the ungenotyped SNPs in these two case cohorts by using phase 3 HapMap and 1000 Genomes pilot project CEU haplotypes as a reference. Since the distributions of the age onset of psoriasis in our four independent case cohorts were not bimodal but positively skewed (Supplementary Figures  1–4), a square-root transformed age onset was used in the analysis. The linear regression quantitative trait test in SNPTEST was used to associate the imputed dosage for each SNP with the age onset of psoriasis separately in GAIN and WashU/UCSF cohorts with the adjustment of gender. Only imputed SNPs with relatively high confidence (PROPER_Info > 0.5) were analyzed [16]. The association results from the GAIN cohort, WashU/UCSF cohort, and UCSF cohort were then combined by meta-analysis using inverse-variance method based on a fixed-effect model. The false discovery rate method was used to correct for multiple testing (FDR_*q* < 0.05). 

## 3. Results

We used linear regression to evaluate the association of 39 psoriasis genetic loci with age of psoriasis onset in a combined sample of 1,498 cases. These loci were identified by previous psoriasis genome-wide association studies and include variants within or near the *HLA* locus, *IL23R, IL12B, TNIP1, TNFAIP3, IL23A, IL13, TRAF3IP2, LCE3B/3C, RNF114, IFIH1, ERAP1, REL, TYK2, NFKBIA, NOS2, IL28RA, SDC4, FBXL19, *and* RPS26*. We found that rs10484554 and rs12191877 near *HLA-C *and rs17716942 near *IFIH1* were associated with age onset of psoriasis with FDR_*q* value < 0.05. As expected, the alleles associated with increased risk of psoriasis were also associated with younger age of onset of psoriasis (Table 1). However, the association between rs17716942 and age of psoriasis onset was not replicated in the NPF cases (beta = −0.01 and *P* = 0.94 for the T allele, Table 1). Since the top two SNPs associated with age of onset were located near the *HLA-C* locus, we further imputed *HLA-C∗06:02* in GAIN, WashU/UCSF, and UCSF case cohorts as previously described [17]. *HLA-C∗06:02* was more strongly associated with age of onset than the two individual *HLA-C* SNPs (Table 1). Our linear regression model revealed that psoriasis patients with two copies of *HLA-C∗06:02* developed psoriasis on average at age 15, psoriasis patients with one copy of *HLA-C∗06:02* developed psoriasis at age 21, and psoriasis patients with zero copies of *HLA-C∗06:02* developed psoriasis at age 27. Thus, each additional copy of *HLA-C∗06:02* reduced the onset of psoriasis by 6 years. 

## 4. Discussion

In this study we found that among the many psoriasis loci discovered by GWAS, only the *HLA-C* locus showed robust evidence of playing a role in earlier psoriasis onset. The *HLA-C∗06:02* allele had a stronger effect on age of onset than individual *HLA-C* SNPs, and this allele was highly significant in each of the three individual cohorts studied (*P* < 0.001). In our study population, psoriasis patients with two copies of *HLA-C∗06:02* developed psoriasis nearly 12 years earlier than those without *HLA-C∗06:02*. This is likely to underestimate the true effect of *HLA-C∗06:02* because our study population contained relatively more type I psoriasis (onset < age 40) than type II psoriasis (onset > age 40) (Supplementary Figures  1–4), where it has been shown that *C∗06:02* is more common in type I psoriasis compared to type II psoriasis [1, 3, 4]. The SNP rs17716942 in the innate antiviral gene *IFIH1* was also significant in the meta-analysis (*P* = 0.002, FDR < 0.05). *IFIH1* encodes a cytoplasmic helicase that acts as a sensor for double-stranded RNA. However, this SNP was not significantly associated with age of onset when examined in an independent cohort, thus further examination of this gene in additional cohorts is warranted. 

Genetic variations in *ERAP1, IL23R,* and *LCE3B/LCE3C* have recently been associated with pediatric-onset psoriasis (onset < age 18) in a small study [18]. However, in that study the same variants were also shown to be associated with adult-onset psoriasis, suggesting, they may not be specific to pediatric-onset psoriasis. In our study, *LCE3B/LCE3C* was significantly associated with age of onset in the GAIN cohort (*P* = 0.007), but not in the meta-analysis (FDR = 0.17). We did not observe any evidence for association of *ERAP1* or *IL23R* in our data. 

Considering that *HLA-C∗06:02* has a much larger effect size (odds ratio > 3.0) on the risk of developing psoriasis compared to the other psoriasis risk alleles (odds ratios < 2.0), it is possible that some of these other risk alleles have a smaller influence on age of onset that would only be detected in a study of larger sample size. There could also be a cumulative additive effect of psoriasis risk alleles on age of onset. Indeed, it has previously been shown that a genetic risk score that combines the genetic burden of the top ten psoriasis genes is significantly higher in patients who develop psoriasis before age 30 compared to those who develop psoriasis after age 30 [19].

In summary, in a large dataset of 1,498 cases we have examined 39 psoriasis variants for their association with age of psoriasis onset. Only the *HLA-C* locus demonstrated robust evidence of association with age of onset, with *HLA-C∗06:02* having the strongest effect. In the meta-analysis a variant in *IFIH1* was also found to be significant, however this SNP was not replicated in an independent cohort. Further studies are needed to clarify the role of genetic variants on the age at which psoriasis manifests. 

## Supplementary Material

The Supplementary Material contains the following: a list of single nucleotide polymorphisms (SNPs) associated with psoriasis that were examined in this study (Supplementary Table 1), a histogram showing the distribution of age of psoriasis onset in the GAIN cohort (Supplementary Figure 1), a histogram showing the distribution of age of psoriasis onset in the Washington University and UCSF cohort (Supplementary Figure 2), a histogram showing the distribution of age of psoriasis onset in the UCSF cohort (Supplementary Figure 3), and a histogram showing the distribution of age of psoriasis onset in the National Psoriasis Foundation cohort (Supplementary Figure 4).

## Figures and Tables

**Table 1:** Meta-analysis of associations between 39 selected SNPs and psoriasis age of onset in three case cohorts.^a^

Nearby gene	Rs_id	Risk allele of psoriasis/effect allele of age onset	*P*_GAIN	*P*_WashU/UCSF	*P*_UCSF	Beta for effect allele of age onset	*P*_heterogeneity	Meta-*P* value	FDR_*q* value	*P*_NPF
HLA-C	HLA-Cw6	—	4.30*E*-14	0.0005	3.61*E*-05	−0.618	0.73	5.28*E*-21	—	—
HLA-C	rs10484554	T/T	2.22*E*-11	0.0167	0.000016	−0.380	0.39	1.87*E*-16	<0.0001	—
HLA-C	rs12191877	T/T	2.79*E*-11	0.0174	0.000018	−0.372	0.39	2.77*E*-16	<0.0001	—
IFIH1	rs17716942	T/T	0.0014	0.0668	0.464	−0.201	0.09	0.002	0.028	0.94
HLA-C	rs13191343	T/T	0.0025	0.9000	−1	−0.188	0.22	0.006	0.054	—
LCE3B-LCE3C	rs4112788	G/G	0.0074	0.8299	0.8248	−0.099	0.37	0.021	0.166	—
FBXL19	rs10782001	G/A	0.0366	0.9682	−1	−0.085	0.37	0.059	0.37	—
SDC4	rs1008953	C/C	0.1876	0.1478	−1	0.104	0.47	0.069	0.37	—
FBXL19	rs12924903	A/G	0.0574	0.8552	−1	−0.074	0.34	0.099	0.37	—
TRAF3IP2	rs240993	T/C	0.0659	0.9254	−1	−0.077	0.40	0.102	0.37	—
IL23R	rs11209026	G/G	0.3062	0.6284	0.1364	−0.163	0.65	0.104	0.37	—
IL12B	rs2546890	A/G	0.0630	0.8726	−1	0.074	0.36	0.104	0.37	—
IL12B	rs12188300	T/T	0.0562	0.5824	−1	−0.110	0.20	0.126	0.409	—
IL12B	rs6887695	G/G	0.1230	0.9613	0.7326	0.065	0.79	0.155	0.466	—
IL23R	rs1004819	—/A	0.3657	0.3654	−1	−0.059	0.66	0.230	0.641	—
IL12B	rs953861	G/A	0.1777	0.8474	−1	0.061	0.45	0.258	0.672	—
RPS26	rs12580100	A/G	−1	0.3151	−1	0.164	−1	0.315	0.768	—
TRAF3IP2	rs13210247	G/G	0.6319	0.0106	−1	−0.052	0.01	0.480	0.985	—
IL28RA	rs4649203	A/A	0.4429	0.9925	−1	−0.039	0.73	0.493	0.985	—
NFKBIA	rs8016947	G/G	0.2424	0.1851	−1	−0.026	0.09	0.572	0.985	—
TRAF3IP2	rs33980500	T/T	0.5199	0.0131	−1	−0.045	0.01	0.573	0.985	—
IL12B	rs3212227	T/G	0.7046	0.7332	0.8764	0.030	0.99	0.606	0.985	—
RNF114	rs2235617	C/G	0.9922	0.9954	0.2331	−0.019	0.55	0.640	0.985	—
IL12B	rs3213094	C/T	0.7599	0.7216	0.8764	0.026	0.98	0.644	0.985	—
REL	rs702873	C/T	0.5496	0.8308	−1	−0.019	0.65	0.658	0.985	—
TRAF3IP2	rs458017	C/C	0.2933	0.0073	−1	−0.034	0.004	0.662	0.985	—
IL23A	rs2066808	A/G	0.3600	0.2295	0.0512	0.040	0.05	0.680	0.985	—
TNIP1	rs1024995	C/C	0.7206	0.1018	−1	−0.027	0.10	0.690	0.985	—
TRAF3IP2	rs13190932	A/A	0.4434	0.0415	−1	−0.021	0.03	0.791	0.985	—
None	rs6809854	G/G	0.6310	0.0975	−1	0.014	0.09	0.794	0.985	—
TRAF3IP2	rs13196377	A/G	0.3915	0.0396	−1	0.019	0.03	0.812	0.985	—
NOS2	rs4795067	G/G	0.7816	0.2546	−1	−0.009	0.25	0.844	0.985	—
RNF114	rs495337	G/A	0.8424	0.9978	−1	0.008	0.93	0.856	0.985	—
IL13	rs20541	G/G	0.7918	0.8979	0.962	0.010	0.97	0.857	0.985	—
NFKBIA	rs12586317	T/C	0.7487	0.7428	−1	0.008	0.67	0.878	0.985	—
ERAP1	rs27524	A/G	0.8240	0.8969	−1	0.007	0.83	0.884	0.985	—
TYK2	rs280519	A/G	0.5867	0.2155	−1	0.004	0.18	0.939	0.992	—
TNIP1	rs17728338	A/A	0.7805	0.6041	0.7386	−0.005	0.80	0.942	0.992	—
TNFAIP3	rs610604	G/T	0.8997	0.9178	0.7863	−0.002	0.95	0.967	0.992	—
TYK2	rs12720356	A/C	0.7275	0.5667	−1	0.001	0.50	0.992	0.992	—

FDR_*q*: false discovery rate adjusted *P* value based on 39 selected SNPs. ^a^In total, 898, 182, 418, and 489 psoriasis cases were included in the GAIN, WashU/UCSF, UCSF, and NPF case cohorts, respectively; for certain SNPs that were imputed with low confidence (proper_info < 0.5 for rs12580100) in GAIN cohort or were not genotyped in UCSF case cohort, their information (denoted as *P* = “−1”) was not included in the corresponding meta-analysis; Cochran's *Q* statistic was used to examine the heterogeneity.
