# Supplementary material for: The Role of 39 Psoriasis Risk Variants on Age of Psoriasis Onset
Source: ISRN Dermatol. 2013 Sep 23;2013:203941. doi: 10.1155/2013/203941 (PMC3794653; doi:10.1155/2013/203941)
Supplement: Supplementary file 1 — The Supplementary Material contains the following: a list of single nucleotide polymorphisms (SNPs) associated with psoriasis that were examined in this study (Supplementary Table 1), a histogram showing the distribution of age of psoriasis onset in the GAIN cohort (Supplementary Figure 1), a histogram showing the distribution of age of psoriasis onset in the Washington University and UCSF cohort (Supplementary Figure 2), a histogram showing the distribution of age of psoriasis onset in the UCSF cohort (Supplementary Figure 3), and a histogram showing the distribution of age of psoriasis onset in the National Psoriasis Foundation cohort (Supplementary Figure 4). [file 203941.f1.zip › Supplementary_Figure_4_DERMATOLOGY_604704.docx]

Supplementary Fig 4. The distribution of age onset in National Psoriasis Foundation subjects (n=489)
