# Supplementary material for: The Role of 39 Psoriasis Risk Variants on Age of Psoriasis Onset
Source: ISRN Dermatol. 2013 Sep 23;2013:203941. doi: 10.1155/2013/203941 (PMC3794653; doi:10.1155/2013/203941)
Supplement: Supplementary file 1 — The Supplementary Material contains the following: a list of single nucleotide polymorphisms (SNPs) associated with psoriasis that were examined in this study (Supplementary Table 1), a histogram showing the distribution of age of psoriasis onset in the GAIN cohort (Supplementary Figure 1), a histogram showing the distribution of age of psoriasis onset in the Washington University and UCSF cohort (Supplementary Figure 2), a histogram showing the distribution of age of psoriasis onset in the UCSF cohort (Supplementary Figure 3), and a histogram showing the distribution of age of psoriasis onset in the National Psoriasis Foundation cohort (Supplementary Figure 4). [file 203941.f1.zip › Supplementary_Table_1_DERMATOLOGY_604700.docx]

**Supplementary Table 1**

Genetic variants associated with psoriasis from genome-wide association studies.^a^

| Chr. | Locus | Base position (hg19) | Nearby gene | SNP | OR^b^ |
| --- | --- | --- | --- | --- | --- |
| 1 | p31.3 | [67670213](http://genome.ucsc.edu/cgi-bin/hgTracks?hgsid=227293831&db=hg19&position=chr1%3A67670213-67670213) | IL23R | rs1004819^1^ | 1.33^1^ |
|  |  | [67705958](http://genome.ucsc.edu/cgi-bin/hgTracks?hgsid=227293831&db=hg19&position=chr1%3A67705958-67705958) |  | rs11209026 (Q381R)^2-4^ | 1.49^2^  0.63^3a^  0.56^4^ |
|  | p36.11 | [24519920](http://genome.ucsc.edu/cgi-bin/hgTracks?hgsid=227293831&db=hg19&position=chr1%3A24519920-24519920) | IL28RA | rs4649203^2^ | 1.13^2^ |
|  | q21.3 | [152551276](http://genome.ucsc.edu/cgi-bin/hgTracks?hgsid=227293831&db=hg19&position=chr1%3A152551276-152551276) | LCE3B-LCE3C | rs4112788^1,2,5^ | 0.76^1^  1.29^2^  1.41^5a^ |
| 2 | p16.1 | [61081542](http://genome.ucsc.edu/cgi-bin/hgTracks?hgsid=228010423&db=hg19&position=chr2%3A61081542-61081542) | REL | rs702873^2^ | 1.12^2^ |
|  | q24.2 | [163260691](http://genome.ucsc.edu/cgi-bin/hgTracks?hgsid=228010423&db=hg19&position=chr2%3A163260691-163260691) | IFIH1 | rs17716942^2^ | 1.29^2^ |
| 3 | p24.3 | [18784423](http://genome.ucsc.edu/cgi-bin/hgTracks?hgsid=228010423&db=hg19&position=chr3%3A18784423-18784423) | None | rs6809854^2^ | 1.14^2^ |
| 5 | q15 | [96101944](http://genome.ucsc.edu/cgi-bin/hgTracks?hgsid=228010423&db=hg19&position=chr5%3A96101944-96101944) | ERAP1 | rs27524^2^ | 1.13^2^ |
|  | q31.1 | [131995964](http://genome.ucsc.edu/cgi-bin/hgTracks?hgsid=228010423&db=hg19&position=chr5%3A131995964-131995964) | IL13 | rs20541^2^ | 1.12^2^ |
|  | q33.1 | [150476004](http://genome.ucsc.edu/cgi-bin/hgTracks?hgsid=228010423&db=hg19&position=chr5%3A150476004-150476004) | TNIP1 | rs1024995^2^ | 1.27^2^ |
|  |  | [150478318](http://genome.ucsc.edu/cgi-bin/hgTracks?hgsid=228010423&db=hg19&position=chr5%3A150478318-150478318) |  | rs17728338^2,6^ | 1.59^6^ |
|  | q33.3 | [158742950](http://genome.ucsc.edu/cgi-bin/hgTracks?hgsid=229354997&db=hg19&position=chr5%3A158742950-158742950) | IL12B | rs3212227^3^ | 0.64^3a^ |
|  |  | [158750769](http://genome.ucsc.edu/cgi-bin/hgTracks?hgsid=228010423&db=hg19&position=chr5%3A158750769-158750769) |  | rs3213094^2^ | 1.39^2^ |
|  |  | [158759900](http://genome.ucsc.edu/cgi-bin/hgTracks?hgsid=228010423&db=hg19&position=chr5%3A158759900-158759900) |  | rs2546890^1^ | 0.65^1^ |
|  |  | [158772582](http://genome.ucsc.edu/cgi-bin/hgTracks?hgsid=228010423&db=hg19&position=chr5%3A158772582-158772582) |  | rs953861^1^ | 1.51^1^ |
|  |  | [158822645](http://genome.ucsc.edu/cgi-bin/hgTracks?hgsid=229354997&db=hg19&position=chr5%3A158822645-158822645) |  | rs6887695^3^ | 0.70^3a^ |
|  |  | [158829527](http://genome.ucsc.edu/cgi-bin/hgTracks?hgsid=228010423&db=hg19&position=chr5%3A158829527-158829527) |  | rs12188300^7^ | 1.70^7^ |
| 6 | p21.33 | [31241109](http://genome.ucsc.edu/cgi-bin/hgTracks?hgsid=228010423&db=hg19&position=chr6%3A31241109-31241109) | HLA-C | rs13191343^7^ | 2.37^7^ |
|  |  | [31252925](http://genome.ucsc.edu/cgi-bin/hgTracks?hgsid=228010423&db=hg19&position=chr6%3A31252925-31252925) |  | rs12191877^1,6,7^ | 2.79^1^  2.64^6^ |
|  |  | [31274555](http://genome.ucsc.edu/cgi-bin/hgTracks?hgsid=228010423&db=hg19&position=chr6%3A31274555-31274555) |  | rs10484554^2,4^ | 4.66^2^  2.8^4^ |
|  | q21 | [111673714](http://genome.ucsc.edu/cgi-bin/hgTracks?hgsid=228010423&db=hg19&position=chr6%3A111673714-111673714) | TRAF3IP2 | rs240993^2^ | 1.25^2^ |
|  |  | [111696091](http://genome.ucsc.edu/cgi-bin/hgTracks?hgsid=228010423&db=hg19&position=chr6%3A111696091-111696091) |  | rs458017^2^ | 1.37^2^ |
|  |  | [111901838](http://genome.ucsc.edu/cgi-bin/hgTracks?hgsid=228010423&db=hg19&position=chr6%3A111901838-111901838) |  | rs13196377^7^ | 1.67^7^ |
|  |  | [111913070](http://genome.ucsc.edu/cgi-bin/hgTracks?hgsid=228010423&db=hg19&position=chr6%3A111913070-111913070) |  | rs13190932^7^ | 1.83^7^ |
|  |  | [111913262](http://genome.ucsc.edu/cgi-bin/hgTracks?hgsid=228010423&db=hg19&position=chr6%3A111913262-111913262) |  | rs33980500 (p.Asp10Asn)^1,7^ | 1.37^1^  1.52^7^ |
|  |  | [111922720](http://genome.ucsc.edu/cgi-bin/hgTracks?hgsid=228010423&db=hg19&position=chr6%3A111922720-111922720) |  | rs13210247^1,7^ | 1.70^1^  1.69^7^ |
|  | q23.3 | [138199417](http://genome.ucsc.edu/cgi-bin/hgTracks?hgsid=228010423&db=hg19&position=chr6%3A138199417-138199417) | TNFAIP3 | rs610604^2^ | 1.22^2^ |
| 12 | q13.2 | [56439209](http://genome.ucsc.edu/cgi-bin/hgTracks?hgsid=228010423&db=hg19&position=chr12%3A56439209-56439209) | RPS26 | rs12580100^8^ | 1.29^8^ |
|  | q13.3 | [56737973](http://genome.ucsc.edu/cgi-bin/hgTracks?hgsid=228010423&db=hg19&position=chr12%3A56737973-56737973) | IL23A | rs2066808^2^ | 1.49^2^ |
| 14 | q13.2 | [35682172](http://genome.ucsc.edu/cgi-bin/hgTracks?hgsid=228010423&db=hg19&position=chr14%3A35682172-35682172) | NFKBIA | rs12586317^8^ | 1.19^8^ |
|  |  | [35832666](http://genome.ucsc.edu/cgi-bin/hgTracks?hgsid=228010423&db=hg19&position=chr14%3A35832666-35832666) |  | rs8016947^2^ | 1.19^2^ |
| 16 | p11.2 | [30928970](http://genome.ucsc.edu/cgi-bin/hgTracks?hgsid=228010423&db=hg19&position=chr16%3A30928970-30928970) | FBXL19 | rs12924903^8^ | 1.15^8^ |
|  |  | [30942625](http://genome.ucsc.edu/cgi-bin/hgTracks?hgsid=228010423&db=hg19&position=chr16%3A30942625-30942625) |  | rs10782001^8^ | 1.16^8^ |
| 17 | q11.2 | [26106675](http://genome.ucsc.edu/cgi-bin/hgTracks?hgsid=228010423&db=hg19&position=chr17%3A26106675-26106675) | NOS2 | rs4795067^8^ | 1.20^8^ |
| 19 | p13.2 | [10469975](http://genome.ucsc.edu/cgi-bin/hgTracks?hgsid=228010423&db=hg19&position=chr19%3A10469975-10469975) | TYK2 | rs12720356^2^ | 1.40^2^ |
|  |  | [10472933](http://genome.ucsc.edu/cgi-bin/hgTracks?hgsid=228010423&db=hg19&position=chr19%3A10472933-10472933) |  | rs280519^2^ | 1.13^2^ |
| 20 | q13.12 | [43980726](http://genome.ucsc.edu/cgi-bin/hgTracks?hgsid=228010423&db=hg19&position=chr20%3A43980726-43980726) | SDC4 | rs1008953^8^ | 1.15^8^ |
|  | q13.13 | [48522330](http://genome.ucsc.edu/cgi-bin/hgTracks?hgsid=228010423&db=hg19&position=chr20%3A48522330-48522330) | RNF114 | rs495337^8,9^ | 1.19^8^  1.25^9^ |
|  |  | [48554977](http://genome.ucsc.edu/cgi-bin/hgTracks?hgsid=228010423&db=hg19&position=chr20%3A48554977-48554977) |  | rs2235617^2^ | 1.20^2^ |

^a^The additional support from previous candidate gene studies were also selected and listed.

^b^ORs are listed in order of preference: meta-analysis studies, replication studies, discovery studies.

References:

1 Ellinghaus E, Ellinghaus D, Stuart PE *et al.* Genome-wide association study identifies a psoriasis susceptibility locus at TRAF3IP2. *Nat Genet* 2010; **42**: 991-5.

2 Strange A, Capon F, Spencer CC *et al.* A genome-wide association study identifies new psoriasis susceptibility loci and an interaction between HLA-C and ERAP1. *Nat Genet* 2010; **42**: 985-90.

3 Cargill M, Schrodi SJ, Chang M *et al.* A large-scale genetic association study confirms IL12B and leads to the identification of IL23R as psoriasis-risk genes. *Am J Hum Genet* 2007; **80**: 273-90.

4 Liu Y, Helms C, Liao W *et al.* A genome-wide association study of psoriasis and psoriatic arthritis identifies new disease loci. *PLoS Genet* 2008; **4**: e1000041.

5 de Cid R, Riveira-Munoz E, Zeeuwen PL *et al.* Deletion of the late cornified envelope LCE3B and LCE3C genes as a susceptibility factor for psoriasis. *Nat Genet* 2009; **41**: 211-5.

6 Nair RP, Duffin KC, Helms C *et al.* Genome-wide scan reveals association of psoriasis with IL-23 and NF-kappaB pathways. *Nat Genet* 2009; **41**: 199-204.

7 Hüffmeier U, Uebe S, Ekici AB *et al.* Common variants at TRAF3IP2 are associated with susceptibility to psoriatic arthritis and psoriasis. *Nat Genet* 2010; **42**: 996-9.

8 Stuart PE, Nair RP, Ellinghaus E *et al.* Genome-wide association analysis identifies three psoriasis susceptibility loci. *Nat Genet* 2010; **42**: 1000-4.

9 Capon F, Bijlmakers MJ, Wolf N *et al.* Identification of ZNF313/RNF114 as a novel psoriasis susceptibility gene. *Hum Mol Genet* 2008; **17**: 1938-45.
